# Age Differences in Affective Outcomes of Daily Emotion Sharing

**DOI:** 10.1093/geroni/igaf122.3528

**Published:** 2025-12-31

**Authors:** Jane Stephenson, Enna Chen, Kathrine Whitman, Laura Carstensen

**Affiliations:** Stanford University, Stanford, California, United States; Stanford University, Stanford, California, United States; Stanford University, Palo Alto, California, United States; Stanford University, Stanford, California, United States

## Abstract

According to socioemotional selectivity theory (SST), as people age and future time horizons shorten, emotionally meaningful goals are prioritized over exploratory ones. Relatedly, people increasingly engage in selective pruning of social networks that focus on emotionally close relationships. Research has not explored the degree to which older adults discuss emotional experiences with close partners and whether potential benefits of emotional disclosures vary by age. An experience sampling study that sampled emotional experiences at five randomly selected times per day for seven days, generated the data analyzed in the present study. A lifespan sample of 180 participants (aged 18 to 93, M = 57.3) reported whether they shared their emotions with someone and, if so, the person with whom they shared, viz., spouse/partner, other family member, friend, acquaintance, coworker, and stranger. They also reported the degree to which they were currently experiencing 8 positive and 11 negative emotions. Although there were no age differences in the likelihood of sharing emotions with any specific partner, sharing emotions with non-spousal family members was associated with more positive emotional experience and less negative emotional experience in older participants than younger participants; and sharing emotions with spouses or romantic partners was associated with relatively more positive experience in younger and middle-aged adults than in older adults. Gender differences and associations with discrete emotions are also discussed. Findings indicate that emotional disclosures in close relationships play different roles at different ages, which have implications for overall well-being.

